# Are we there yet? A systematic literature review of Open Educational Resources in Africa: A combined content and bibliometric analysis

**DOI:** 10.1371/journal.pone.0262615

**Published:** 2022-01-18

**Authors:** Ahmed Tlili, Fahriye Altinay, Ronghuai Huang, Zehra Altinay, Jako Olivier, Sanjaya Mishra, Mohamed Jemni, Daniel Burgos

**Affiliations:** 1 Smart Learning Institute of Beijing Normal University, Beijing, China; 2 Near East University, Institute of Graduate Studies, Faculty of Education, Societal Research and Development Center, Nicosia, North Cyprus, Mersin, Turkey; 3 Research Unit Self-Directed Learning, Faculty of Education, North-West University, Potchefstroom, South Africa; 4 Commonwealth of Learning, Vancouver, Canada; 5 Arab League Educational, Cultural and Scientific Organization, Tunis, Tunisia; 6 Research Institute for Innovation & Technology in Education (UNIR iTED), Universidad Internacional de La Rioja (UNIR), Logroño, Spain; National Taiwan University of Science and Technology, TAIWAN

## Abstract

Although several studies have been conducted to summarize the progress of open educational resources (OER) in specific regions, only a limited number of studies summarize OER in Africa. Therefore, this paper presents a systematic literature review to explore trends, themes, and patterns in this emerging area of study, using content and bibliometric analysis. Findings indicated three major strands of OER research in Africa: (1) OER adoption is only limited to specific African countries, calling for more research and collaboration between African countries in this field to ensure educational equity; (2) most of the OER initiatives in Africa have focused on the creation process and neglected other important perspectives, such as dissemination and open educational practices (OEP) using OER; and (3) on top of the typical challenges for OER adoption (e.g., infrastructure), other personal challenges were identified within the African context, including culture, language, and personality. The findings of this study suggest that more initiatives and cross-collaborations with African and non-African countries in the field of OER are needed to facilitate OER adoption in the region. Additionally, it is suggested that researchers and practitioners should consider individual differences, such as language, personality and culture, when promoting and designing OER for different African countries. Finally, the findings can promote social justice by providing insights and future research paths that different stakeholders (e.g., policy makers, educators, practitioners, etc.) should focus on to promote OER in Africa.

## 1. Introduction

The African continent is diverse–culturally, socio-economically, and in terms of approaches to education and availability of educational technologies. The continent comprises 54 countries, and it is the second-largest continent [1]. Open Educational Resources (OER) are deemed useful in under-resourced areas within the continent. In addition, the affordances of OER are apparent in that they provide possibilities toward decolonizing the curriculum by providing adaptable resources [2]. This aspect is quite important in economically poor contexts or contexts whose educational systems still draw on content from former colonial powers. According to the Recommendation on OER by UNESCO [3], OER are “learning, teaching and research materials in any format and medium that reside in the public domain or are under copyright that have been released under an open license, that permit no-cost access, re-use, re-purpose, adaptation and redistribution by others” (p. 5). The use of OER in educational contexts also involves what is termed Open Educational Practices (OEP).

In the context of Africa, the concept of OER has been embraced by several organizations and educational institutions, yet the practices and levels of adoption vary a lot. In 2014, Seychelles adopted ICT in education and training policy that for the first time recognized OER in any African country at the national level to improve access to quality learning materials [4]. Nigeria adopted a national OER policy for higher education in 2017 prior to the second World OER Congress. Some institutions–like the Africa Nazarene University, Botswana Open University, Kwame Nkrumah University of Science and Technology, the National Open University of Nigeria, the University of Cape Town, and the University of South Africa–have OER policy supporting the use, creation, and sharing of teaching and learning materials with an open license.

In Africa, as with most of the Global South, OER could potentially contribute to solutions for distinct educational challenges. The latter include “unequal access to education; variable quality of educational resources, teaching and student performance; and increasing cost and concern about the sustainability of education” [5]. Therefore, it is necessary to consider what research has been conducted on OER in the African context in order to identify trends. It is, however, also necessary to determine research lacunae for future work. Though several studies have been conducted to understand the current situation of OER in a given region (e.g., [6, 7], limited information could be found that summarized OER progress in Africa. Therefore, to promote social justice and facilitate OER adoption in the African context, a systematic literature review was undertaken. To the best of our knowledge, no systematic review has been conducted on OER in Africa–hence, the reported findings on this field are still divided. Consequently, it remains difficult to identify research gaps and future research on the ways of facilitating OER adoption in Africa.

This systematic review entailed both bibliometric and content analysis. Bibliometric analysis was adopted because it provides visual representations of the relationships between the main concepts [8]. This visualization through mapping allows researchers to identify the background of a given research field, the relationships between key concepts, and possible future trends [9]. On the other hand, content analysis was adopted to acquire an in-depth analysis of the reviewed studies–hence, to identify research themes that authors focused on while discussing OER in Africa. This systematic review answers each of the following research questions that elicits valuable theoretical and practical implications on this topic of interest. On one hand, cross-country/culture distribution of studies helps evaluate the scientific perspective and innovativeness of countries in adopting OER. This can help creating an OER roadmap to support those countries lacking behind and establishing cross-collaborations with countries which are leading the field for improved regional equality related to OER adoptions. Also, the identified trends across years of publications, publication venues and themes allow the assessment of OER research development. For instance, the publication frequency per year is a valuable indicator of the complexity and maturity of research on the topic, while are also closely related to the industrial knowledge, skills, experience, and training adequacy in the corresponding direction [10]. The chosen publication venue can reflect the followed publication policy by researchers in the field, as if researchers are targeting open-access journals in line with the OER concept or not. The covered OER research themes through the keywords used or content, provide current research paths of adopting OER, thereby enriching our understanding of the landscape of this research area thus far [11]. Additionally, the different research methods identified in each publication reflect the nature of each study conducted on the topic (e.g., conceptual vs. empirical, practice-oriented or scholarly-oriented, etc.). Finally, identification of OER limitations can help providing guidelines of OER adoption in different countries. In this study, the following research questions were answered:

**RQ1.** How have publications on OER in Africa evolved over time, from which countries/regions, and in which publication venue?
**RQ2.** What are the research methods used in the studies about OER in Africa?
**RQ3.** What initiatives have been launched to facilitate OER adoption in Africa?
**RQ4.**What are the most frequently used terms in the keywords, abstracts, and titles of OER-in-Africa studies?
**RQ5.**What are the OER in Africa themes that researchers have focused on?
**RQ6.**What are the challenges that limit OER adoption in Africa?

## 2. Method

### 2.1. Research method and design

In this study, a “mixed-methods systematic review,” termed by [12], which combines quantitative and qualitative synthesis approaches was used. A traditional systematic review is an important step before carrying out any study. It builds the foundation for knowledge accumulation, which, in turn, facilitates the expansion and improvement of theory, closes gaps existing in research, and uncovers areas previous research has missed [13, 14]. However, outcome reporting bias may be introduced, and the interpretation of results is prone to be subjective in a manual review [15]. Therefore, a mixed-methods systematic review that combines bibliometric analysis and content analysis is needed to scientifically identify the knowledge base and evolution of a topic [16, 17]. a systematic review was conducted based on the published studies related to OER in Africa. The Preferred Reporting Items for Systematic Reviews and Meta-Analyses (PRISMA) guidelines were followed to produce this systematic review [18]. PRISMA provides a standard peer-accepted methodology that uses a guideline checklist, which was strictly followed.

As part of the review, content analysis [19], and bibliometric analysis [8] were used. The data were analyzed and interpreted through these approaches for the purpose of data triangulation in order to gain a multi-dimensional perspective [20] and increase the validity of the research. For the bibliometric analysis and synthesis, VOSviewer software was used to make distance-based co-occurrence maps: terms retrieved from keywords, titles, and abstracts were clustered and mapped according to their relatedness in a similarity matrix [21]. The software was also used for co-authorship analyses of the studies.

### 2.2. Search strategy and selection criteria

To deal with this complex topic, an extensive search for research papers and articles was conducted using the following search strings. Each of the following strings or substrings are popular keywords and synonyms used in the literature when discussing “open educational resources” or “Africa”:

*Search string*: (open educational resources) AND (Africa)

*Open educational resources substring*: open educational resources OR open educational practices OR open learning OR open education

*Africa substring*: Africa OR Sub-Saharan Africa OR Global South.

Moreover, the search was conducted in the following databases: Scopus, ScienceDirect, and Google Scholar. These are some of the largest databases of peer-reviewed scholarly literature [22, 23]. After searching the relevant databases, two coders analyzed the retrieved papers by title, abstract and, if necessary, by full text, based on pre-defined inclusion and exclusion criteria. An article was excluded if (1) the full text was not available online; (2) it was not in English; (3) it discussed openness in other fields (e.g., health, agriculture, etc.); (4) OER and OEP were not investigated or discussed; and (5) it provided superficial findings without any useful insights. Finally, based on the degree of agreement between the choices made by the two independent coders in selecting papers, Cohen’s kappa was calculated to test inter-rater reliability. According to [24], inter-rater reliability was very good (κ = 0.81), where in case the assessment score was different, agreement was reached through discussions.

### 2.3. Quality assessment

To assess the overall quality of each selected study, five criteria were used, with each criterion focusing on a different quality issue. Particularly, each Quality Criterion (QC) was a yes/no question, as presented below, corresponding to a score of 1 or 0, respectively.

QC1. Did the study report the sources and details of outcome assessment?
QC2. Did the study compare its reported results with previous results?
QC3. Did the study conduct validity or reliability tests during the quantitative analysis?
QC4. Did the study involve a statistical analysis of significance during the quantitative assessment?
QC5. Did the study report detailed descriptions about the involved groups (e.g., control and experimental group)?

The final score of a given study was the average of the five achieved scores across criteria. Numerous studies have used similar strategies for assessing quality, such as in [25–27].

### 2.4. Data extraction

It is important to note that coding in systematic reviews differs from primary research, as it is conducted by interpreting both the participant data and author analysis to provide third-order constructs [28]. In this systematic review, each of the research elements was coded individually in order to answer each of the above research questions. Descriptive data of each study–including publication year, author list, and affiliation–were identified at first.

As regards the research method, the majority of open and distance education research can be broadly classified as quantitative, qualitative, or mixed design (triangulation); the latter employs both quantitative and qualitative approaches [29]. For this review, the research methods were classified as quantitative, qualitative, mixed, or other. Articles that contained statistical analysis were classified as quantitative, from simple methods such as chi-square analysis to multivariate techniques. Qualitative studies were data-based articles that did not quantify data beyond frequency counts. Other articles were usually descriptive, not data-based, theoretical papers. Sample size was also extracted in each study.

To classify the OER initiatives highlighted in the reviewed studies, the OER initiative framework by [30] was used. This framework contains four categories: creation of OER; organization of OER; dissemination of OER; and utilization of OER.

Finally, the themes and challenges of each study were open-coded to identify important words or groups of words from the data. It was an iterative, inductive process. The codes were deemed to be theoretically saturated once all the research purposes fit into one of the categories.

A qualitative synthesis was conducted to answer the research question. Qualitative syntheses are used in different areas, including Computer Science, for example, in Software Engineering [31]. The next subsection presents the synthesis of the systematic review based on the quality criteria and data extracted from the selected papers.

### 2.5. The final selected papers

**Fig 1:**
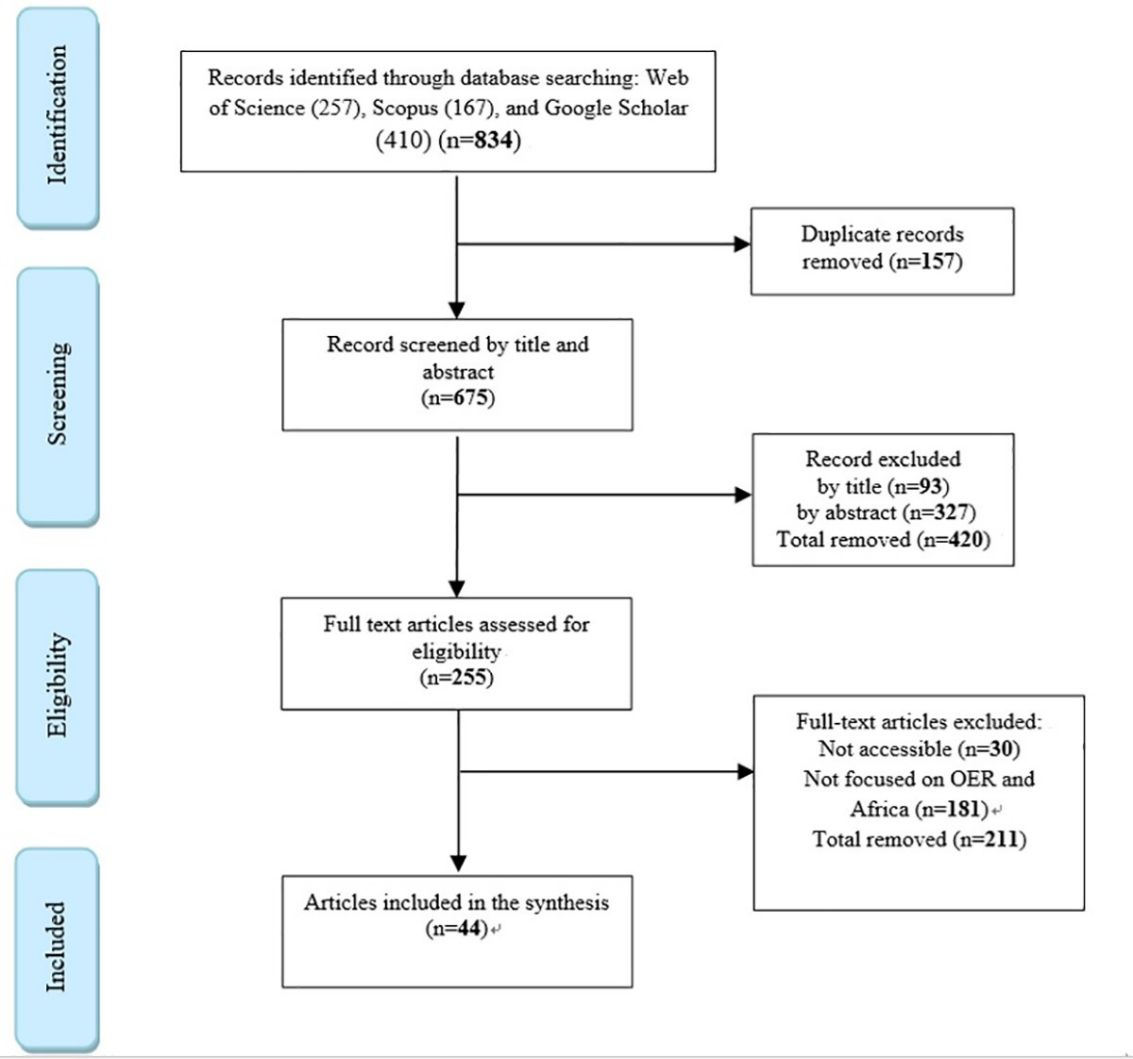
Flowchart of the systematic review process.

This search yielded a total of 834 articles. After discarding duplicated papers, 675 papers remained. Based on screening of title and abstract, 420 papers were then removed. The remaining 255 papers were considered and assessed as full texts. Of these papers, 211 did not fulfil the inclusion criteria, thus a total of 44 eligible research studies remained for final review. Fig 1 presents the study selection process as recommended by the PRISMA group [18]. The dataset used in this study is published under an open-license on *Figshare* repository (please see: https://figshare.com/articles/dataset/OER_in_Africa_dataset_xlsx/17021738).

Table 1 shows the quality scores of the 44 selected studies based on the five quality criteria described above (see Quality Assessment section). If the QC was available, a value of 1 was given in the corresponding cell of Table 1, otherwise a value of 0 was given. Finally, the quality score of each paper across the five QC is presented in the last column. As shown in Table 1, three papers obtained a very high-quality score (more than 0.75) using the selected criteria. Eleven papers obtained a medium score (between 0.5 and 0.75), whereas 30studies obtained a low-quality score less than 0.5. Specifically, most of the studies had reported the sources and details of outcome assessment, 16 studies conducted a statistical analysis of significance during the quantitative analysis, and three studies used valid and reliable outcome measures. Most of the studies (39 out of 44) compared their reported results with previous results, but none of the studies reported a detailed description of the involved groups. It is also seen that all the studies fulfilled at least one QC.

**Table 1:** The quality scores of the selected papers.

Studies	QC1	QC2	QC3	QC4	QC5	Quality Score
[32]	1	1	0	0	0	0.4
[33]	1	1	0	0	0	0.4
[34]	1	1	0	1	0	0.6
[35]	1	1	0	1	0	0.6
[36]	1	1	0	1	0	0.6
[37]	1	1	0	0	0	0.4
[38]	1	1	0	1	0	0.6
[39]	1	1	0	0	0	0.4
[40]	1	1	0	1	0	0.6
[41]	1	1	0	0	0	0.4
[42]	1	1	0	0	0	0.4
[43]	1	1	0	0	0	0.4
[44]	1	1	0	0	0	0.4
[30]	1	1	0	0	0	0.2
[45]	1	1	0	1	0	0.6
[46]	1	1	0	0	0	0.4
[47]	1	1	1	1	0	0.8
[48]	0	0	0	1	0	0.2
[49]	1	1	0	0	0	0.4
[50]	1	1	1	1	0	0.8
[51]	1	1	0	1	0	0.4
[52]	1	1	0	0	0	0.4
[53]	1	1	0	0	0	0.4
[54]	1	1	0	0	0	0.4
[55]	1	1	0	0	0	0.4
[56]	1	1	0	0	0	0.4
[57]	1	0	0	0	0	0.2
[58]	1	1	0	0	0	0.4
[59]	1	1	0	0	0	0.4
[60]	1	1	1	1	1	1
[61]	1	1	0	1	0	0.6
[62]	1	1	0	1	0	0.6
[63]	1	1	0	1	0	0.6
[64]	1	1	0	1	0	0.6
[65]	1	1	1	1	0	0.8
[66]	1	1	0	0	0	0.4
[67]	1	1	0	0	0	0.4
[68]	1	1	0	0	0	0.4
[69]	1	1	0	0	0	0.4
[70]	1	1	0	1	0	0.6
[71]	1	1	0	1	0	0.6
[72]	1	1	0	0	0	0.4
[73]	0	1	0	1	0	0.4
[74]	1	1	0	1	0	0.6

## 3. Results and discussion

### 3.1. How publications on OER in Africa have evolved over time, from which countries/regions, and in which publication venue

**Fig 2:**
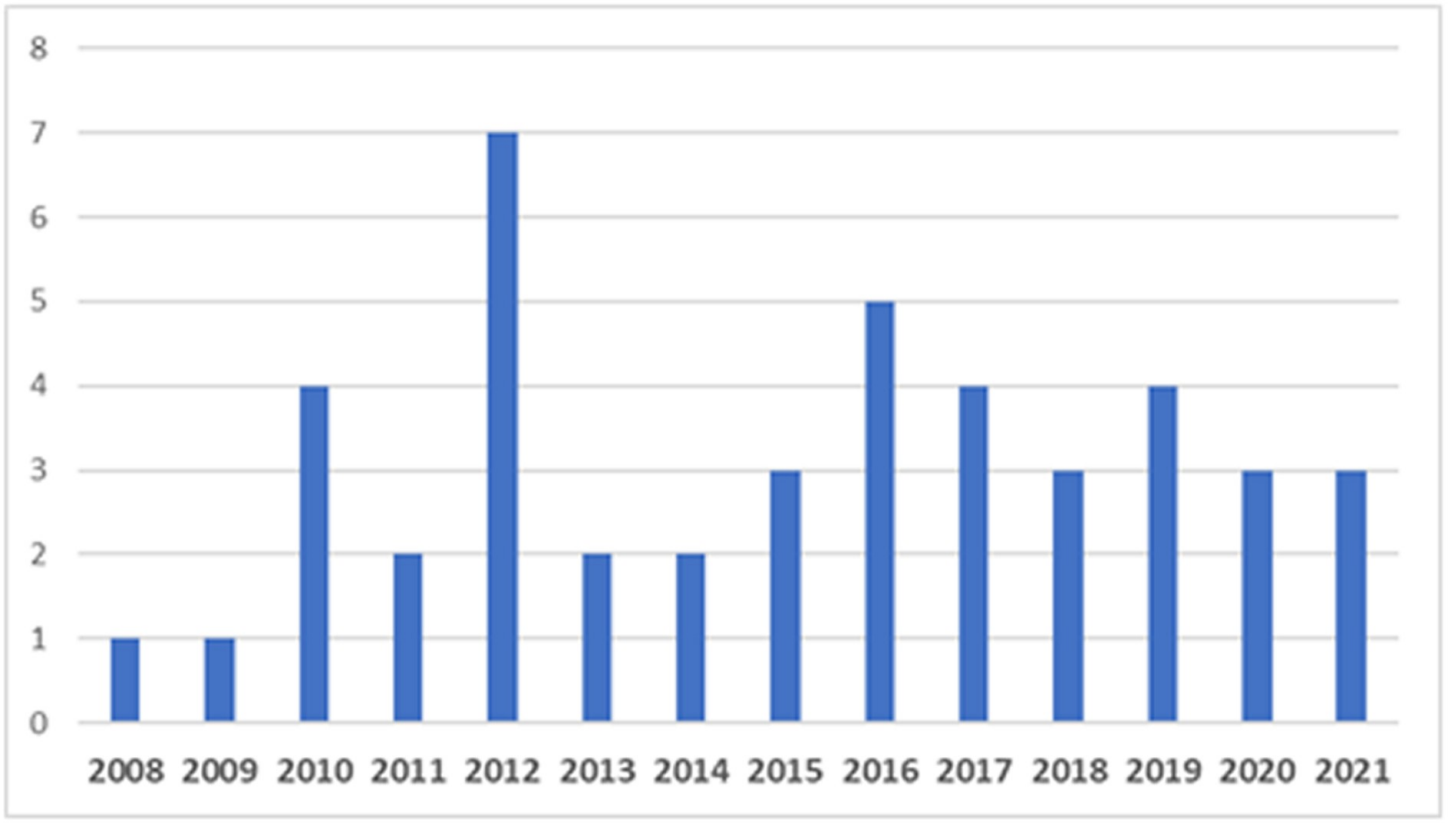
Study distribution by publication year.

Based on the reviewed studies, it is seen that the work on adopting OER in Africa, within the publications included in the analyzed corpus, started in 2008, as shown in Fig 2. One possible reason can be the activities by the William and Flora Hewlett Foundation and the Shuttleworth Foundation in Africa during that period through which work on OER was supported, such as funding for OER Africa and others [39, 75, 76]. This is part of the Hewlett Foundation’s ongoing support for OER initiatives worldwide since 2002 [77]. The Hewlett Foundation has been a key supporter of the open movement, donating over US$170 million over the past 20 years. At that time, around 2008, Hewlett’s overall goals focused on the promotion of free, useful educational materials for all, including Africa. The foundation supported many types of OER from different nations and parts of the world, such as OCW, full courses, teacher training, textbooks, lessons, and simulations [75]. During this period, OER Africa–a project of the South African Institute for Distance Education–was launched in Nairobi to support local OER communities across the African continent.

However, the highest pick was in 2012, as shown in Fig 2. This pick could be explained by the official adoption of OER during the World Congress in 2012, Paris Declaration [78], where several international researchers started paying more attention to this concept, including in Africa. Therefore, it can be concluded that OER policies and initiatives were catalysts for OER research and application in Africa.

These OER research studies were published in different venues. Most of them were published in the *International Review of Research in Open and Distributed Learning* (12 documents), followed by *Open Praxis* (five documents) and, finally, both the *Journal of Interactive Media in Education* and *Distance Education* (two documents each). This could be explained by the fact that these journals allow open-access publishing (at no charge), which is consistent with OER principles. On the other hand, most conferences do not provide this possibility. We also found that *International Review of Research in Open* and *Open Praxis* were the most frequently targeted journals for OER-in-Africa studies. These journals could have been chosen for two reasons: (1) these journals are fully open access with no article processing charge and free for both authors and readers; and (2) they are supported by well-known editors or publishers in the field of OER. For instance, *Open Praxis* is published by the International Council for Open and Distance Education (ICDE), whereas the editor of *International Review of Research in Open and Distance Learning* is Rory McGreal–a leading researcher in the field of open education (including OER).

**Fig 3:**
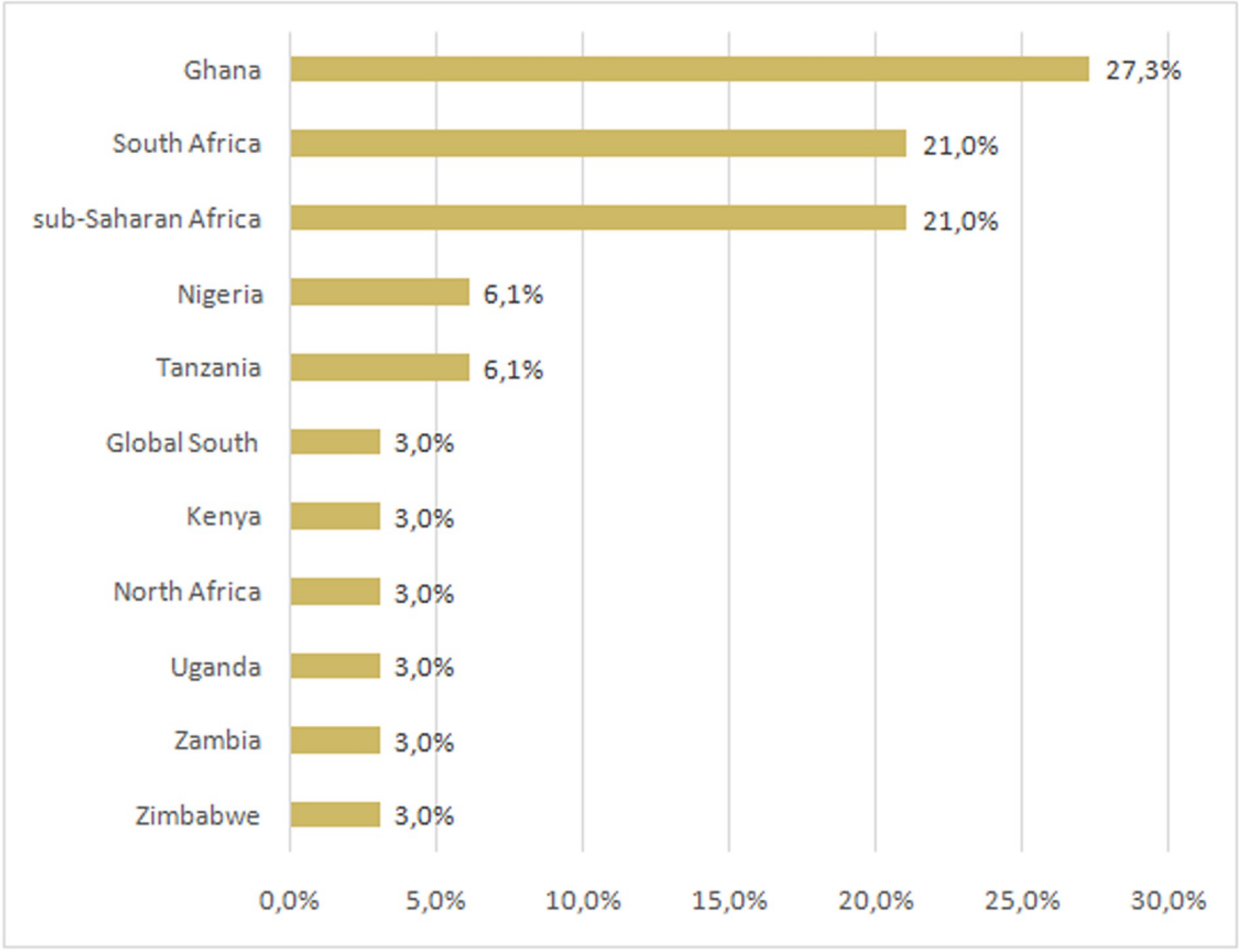
Clustered bar chart with country-of-focus distribution.

Furthermore, studies on OER in Africa have focused on specific countries like Ghana or South Africa and also on an entire region like Sub-Saharan Africa or North Africa, as shown in Fig 3. It is seen that 27.3% and 21% of the studies investigated and discussed OER in Ghana and South Africa, respectively, scoring the highest value. It is evident that open practices are aligned with existing culture within education in terms of sharing and adapting resources [36, 39, 68]. As regards research and projects on OER, South Africa has been at the forefront of activities in this context, with a number of organizations and institutions working toward OER use and practices [69]. According to [66] and Mays (2020), not only does South Africa have a good policy environment in support of OER, but has also seen a number of resources that have been the source of academic enquiry. From the region perspective, 21% of the studies focused on investigating and discussing OER in the Sub-Saharan Africa region. This aspect ties in with ongoing interest in OER within the context of open practices and research in this region [76].

Additionally, the studies came from 29 countries. Table 2 shows the top countries that contributed to at least two documents. South Africa had the most published OER studies (thirteen documents), followed by the United Kingdom (six documents), Ghana (five documents), and Kenya (four documents).

**Table 2:** Top countries contributing to the literature on OER in Africa.

Country	Number of studies
South Africa	13
United Kingdom	6
Ghana	5
Kenya	4
Germany	3
Nigeria	2
China	2
Canada	2

**Fig 4:**
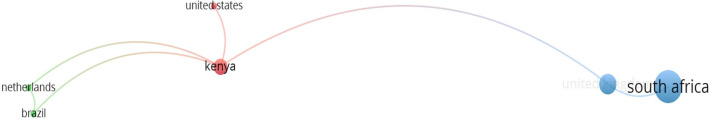
Visualization of co-authorships by country.

Fig 4 presents the co-authorship network by country. The colors represent cooperation clusters. This means that countries belonging to one cluster (shown in the same color) have cooperated more with each other than with countries in other clusters. The size of the circles represents the number of publications from that country. For instance, South Africa has the biggest circle because it yielded the most publications (see Table 2). The lines stand for the co-authorship relationship. The thicker the line, the more co-authorships exist between the two countries linked by it. Fig 4 shows that several African countries have cooperated with several non-African countries while investigating OER in Africa. For instance, South Africa and the United Kingdom (the name tag was hidden by the software because the two circles overlap) have worked together in this field. This type of collaboration can be expected due to increased opportunities for outside funding or drawing in external expertise. However, this is significant, as in terms of OER practices, research has shown that generally, teachers at both school and higher education level tend to work autonomously rather than collaboratively, unless they are part of a project requiring collaboration [60]. It also shows that Kenya has cooperated with the United States, the Netherlands, and Brazil.

### 3.2. Research methods used in the studies on OER in Africa

**Fig 5:**
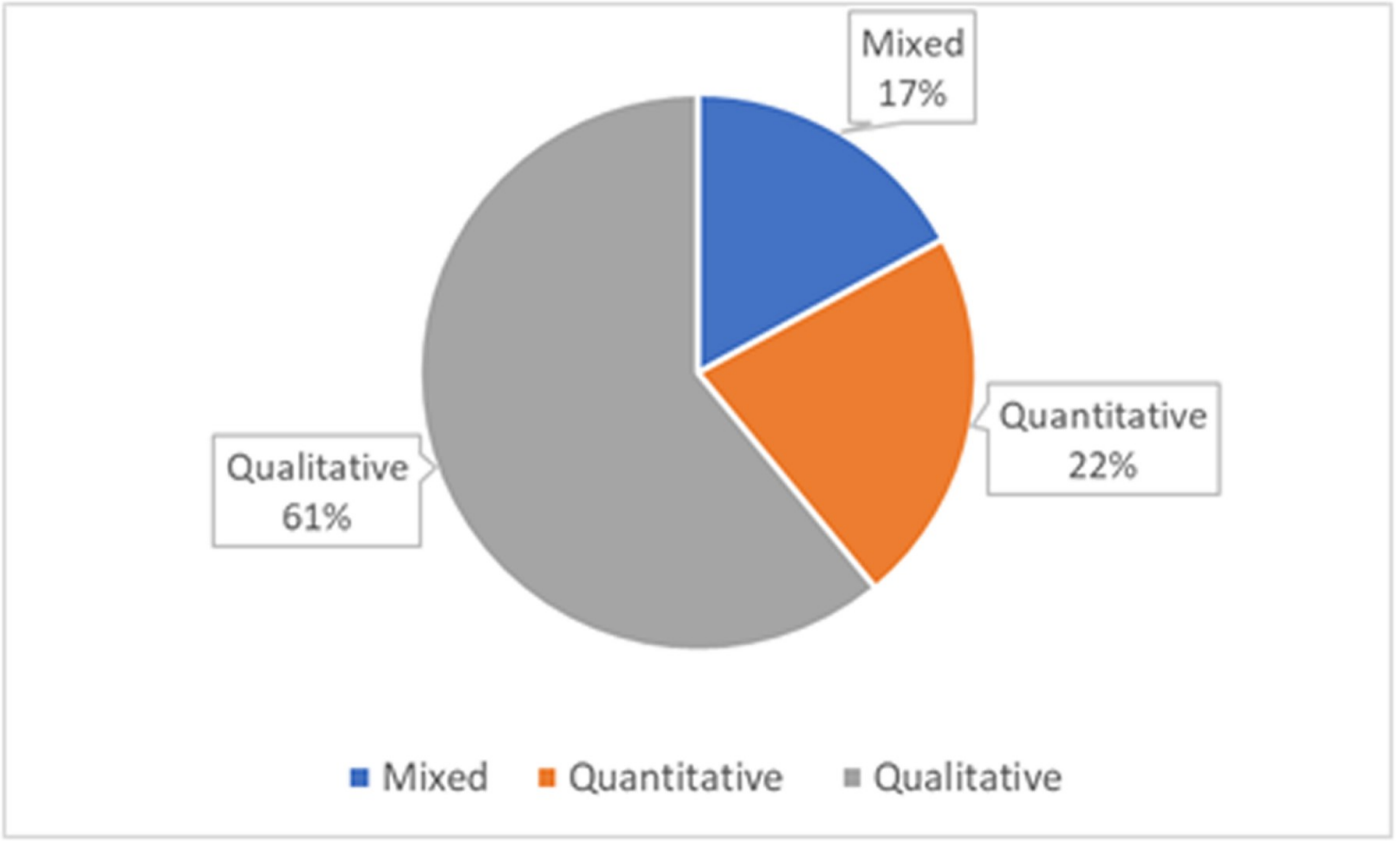
Distribution of research methods in OER-in-Africa studies.

As shown in Fig 5, 61% of the studies on OER in Africa used qualitative methods by analyzing different interviews collected from different stakeholders (e.g., teachers, students, etc.) or while conducting a content analysis of a literature review. On the other hand, 22% of the studies on OER in Africa have used quantitative methods by statistically analyzing the results of different designed surveys. Finally, 17% were mixed methods studies that focused on triangulating the results from surveys and interviews to gain more insight. Additionally, it was found that more than 80% of the studies conducted experiments with a limited sample size (less than 50 participants).

Interestingly, most of the studies relied on surveys and interviews; these can only obtain students’ subjective perceptions which can be biased or faked easily [79]. However, no study focused on analyzing students’ learning traces or behaviors to gain more concrete insight into how different stakeholders (e.g., teachers or students) used or perceived OER. In this context, it is suggested that open data, openly licensed, interoperable, and re-usable datasets be designed and used in the African context to learn more about how OER is being used and perceived in different universities and in African countries. It is stated [80] that open data could be crucial in effectively analyzing and interrupting data to evaluate a given project.

### 3.3. Initiatives that have been launched to facilitate OER adoption in Africa

Table 3 presents the initiatives that were extracted from the reviewed studies, as well as their categories, collaborators, and objectives. Most of these initiatives focused on creating OER; however, less attention has been paid to the other dimensions that can facilitate OER adoption in Africa, such as dissemination. This implies that if OER are created but people do not know about them (e.g., where they are located, how they can be accessed, etc.), they still cannot find and use them. Only two initiatives focused on disseminating OER via, for instance, the “OER Africa” repository. Additionally, most of the initiatives were launched in collaboration with leading foreign institutes or organizations (e.g., Hewlett foundation, University of Michigan, etc.). African governments have made less effort toward OER. Furthermore, none of the initiatives focused on promoting the implementation of OEP using OER for better learning experiences and outcomes [81].

**Table 3:** Summary of OER initiatives in Africa.

Category	Initiative	Collaborators	Objective
**Creation**	TESSA (Teacher Education in Sub-Saharan Africa)	Open University in the UK managed through 18 partner institutions across Sub-Saharan Africa	To develop and produce a large bank of highly structured OER to support teacher education; in each of five primary school curriculum subject areas, there are three modules, each with five sections (a total of 75 sections)
**Creation**	African Health OER Network	OER Africa and the University of Michigan	To provide tools for the conversion of teaching resources to OER
**Creation**	GENIE programme,	National Laboratory of Digital Resources of the Ministry of Education, Morocco	To validate and give certification to digital resources that are in development
**Creation and Dissemination**	Saide’s African Storybook Initiative	SAIDE (South African Institute for Distance Education)	To support and promote literacy in the languages of Africa using digital storybooks made available through openly licensed digital storybooks distributed by means of web-based Internet and mobile app services.
**Dissemination**	OER Africa (OERA)	William & Flora Hewlett Foundation	To play a leading role in driving the development and use of OER in Africa and to provide a common conceptual framework for SAIDE OER-related activities; to harness African experts and expertise; and to deploy OER to the benefit of Africa’s higher education system
**Utilization**	University of Cape Town (UCT)	Center for Educational Technology & Faculty of Health Sciences	To aid academics in converting content into OER

### 3.4. Frequently used terms in the keywords, abstracts, and titles of OER-in-Africa studies

**Fig 6:**
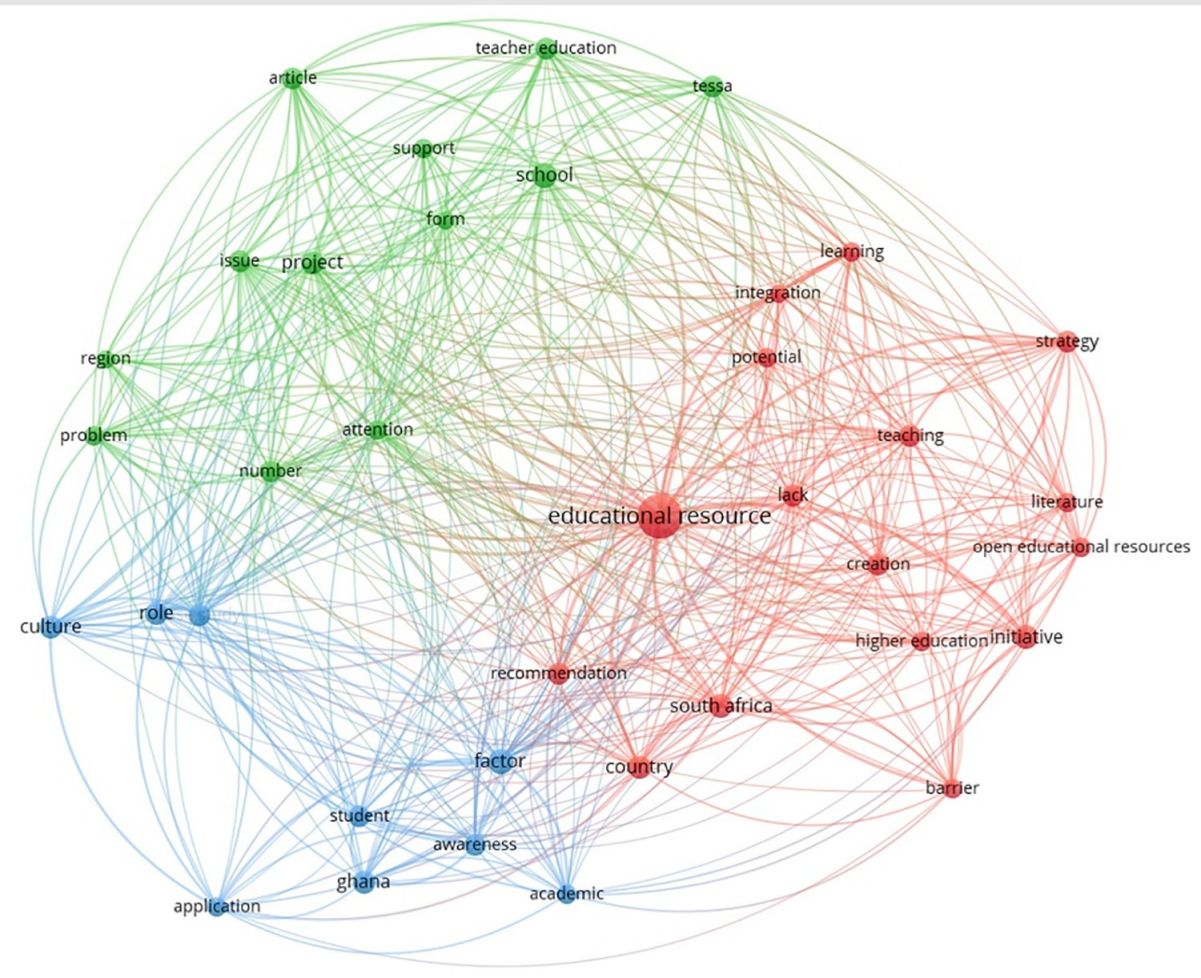
Co-occurrence network map of terms found in titles and abstracts.

To identify the focus and trends of OEP research, the co-occurrence of terms in both abstracts and titles was analyzed using binary counting. The threshold for including a term in the analysis was a minimum of five occurrences. Out of 1172 terms, only 62 terms met this threshold. However, only 37 terms were selected and considered based on a relevance score calculated by VOSviewer. The relevance score is useful for identifying the more informative terms that better represent specific topics [82]. The resulting co-occurrence network map is shown in Fig 6; three final clusters are presented in different colors (red, green, and blue). The red cluster seems to focus on the different discussed OER topics, with terms like “open educational resources”, “educational resources”, “initiative”, “creation”, “integration”, and “barrier”. The green cluster seems to focus on the context, covering terms like “school”, “teacher education”, “region”, and “TESSA”. Finally, the blue cluster seems to focus on the different factors that might affect OER adoption, with terms like “culture”, “awareness”, “academic”, “role”, and “factor”.

The size of the labels and circles depends on the number of occurrences. Lines identify major links between terms, and their thickness and the distance between the terms represent the association strength. For example, in Fig 6, the terms “educational resources” and “lack” have a short distance between them, which means that they occurred together several times. One of the reasons for adopting OER in Africa is thus to increase accessibility to educational resources, which is a major problem in several African countries [69, 76]. The distance is also very short between the terms “initiative” and “higher education”, meaning that most of the initiatives toward OER adoption in Africa focused on higher education instead of other educational levels such as K-12 or vocational education. Therefore, more focus should also be on those educational contexts to investigate how teachers and students would design their learning materials accordingly. Interestingly, several studies focused on the cultural perspective when discussing OER in Africa. This might be ascribed to several African educators still believing that high-quality content cannot be open and created by anyone [74].

### 3.5. The OER themes researchers have focused on

Analysis of the OER research focus in Africa (see Table 4) indicated that the main goal was to enhance education (69.12%). Particularly, in this field, researchers have focused on improving educational resources (23.53%) and teacher education (4.41%). For instance, many countries in Sub-Saharan Africa make use of distance education and school-based learning approaches for both the initial and continuing education of teachers while in service. This means that many institutions offering teacher development have already made a move toward resource-based forms of provision and logically should have a natural inclination to engage with OER as providing a way to strengthen curriculum offerings and to be able to adapt existing content for a better fit with different contexts [68]. [78] put emphasis on the quality concerns of OER in determining materials. In addition [40], stated the importance of reflective action for encouraging adapting and adopting best practices in OER.

**Table 4:** Summary of the OER research focus in Africa.

Themes and Subthemes	Frequency (f)	Percentage (%)
**Attitudes and intentions toward OER adoption**	**12**	**8.82%**
Academicians’ and lecturers’ adaptation	8	5.88%
Adoption, openness, reflective–Mindset change	2	1.47%
Information and communication technologies adaptation	2	1.47%
**Challenges**	**10**	**7.35%**
Access challenges	8	5.88%
OER adoption challenges	2	1.47%
**Culture**	**11**	8.09%
Cultural context	9	6.62%
Institutional OER culture	2	1.47%
**Education**	**94**	**69.12%**
Access to education	5	3.68%
Educational resources	32	23.53%
Enhance quality education	15	11.03%
Equity (gender &culture & socio-economical)	1	0.74%
Health education OER	2	1.47%
Higher education	14	10.29%
Pedagogical	4	2.94%
Professional development	2	1.47%
Sustainability	5	3.68%
Teacher education	6	4.41%
TESSA	8	5.88%
**Policy**	**9**	**6.62%**
Governmental practices	1	0.74%
OER policies	8	5.88%
**Total**	**136**	100.00%

Speaking of educational equity, several studies focused on discussing how OER can reduce cost and facilitate access to education [66, 68], especially as in some African countries, 12 children share one reading textbook, and 14 children have access to one math textbook in school [83]. One study highlights that enhancing access to teaching, learning and research information resources to institutions [35]. On the other hand, the study of [66] underlines the access, collaboration, and transformation, as three dimensions to democratize access to education through OER.

It was also found that 8.82% of the studies focused on attitudes and intentions toward OER adoption. For instance [74], focused on how the perception of Ghanaian educators toward OER adoption varied depending on many international and external factors. In the latter study, the findings indicated that whether and how OER adoption takes place at an institution are shaped by a layered sequence of factors such as infrastructural access, legal permission, conceptual awareness, technical capacity, material availability, and individual or institutional volition that are further influenced by prevailing cultural and social variables [55]. One study of [63] provided insights on the awareness and appreciation of the OER concept, and open licensing is low, but there is a ready step for openness in the future.

However, although several scholars and organizations suggest that policies are a key factor for catalyzing the process of OER adoption, it was found that only 6.62% of the studies focused on discussing OER policies in Africa. One study of [71] emphasized the OER policy-making issue for achieving social justice. One example of this is UCT. In contrast to most other universities in the country, UCT academics are allowed to possess the copyright of their teaching materials and thus turn them into OER. The UCT IP Policy states that UCT automatically assigns to the author(s) the copyright with the provision that UCT retains a perpetual, royalty-free, non-exclusive license to use, copy, and adapt such materials at UCT for teaching and/or research purposes. However, given the diversity of institutions in the higher education sector and the administrative and financial challenges facing many institutions in Africa, it might not always be clear which type of policy would work best in a given context. Some policies might simply act as a “hygienic” factor that is a necessary but not a sufficient variable in promoting OER activity, whereas others might act as a “motivating” factor that is incentivizing OER activity, either among individual academics, or the institution as a whole [47].

### 3.6. Challenges that limit OER adoption in Africa

As shown in Table 5, several types of challenges were identified from the reviewed studies. Most of the studies shed light on OER policies as a limitation: lack of governmental and institutional policies are factors that limit OER adoption in Africa. For instance [50], stated that lack of non-compliant policies and incentives, lack of OER accreditation, and cataloging standards limit OER adoption in Africa.

**Table 5:** Challenges to OER in Africa.

Dimensions	Factors
Policies	1. Lack of governmental and institutional policies 2. Lack of copyright laws 3. Lack of OER awareness 4. Lack of policy making
Infrastructure	1. Lack of ICT infrastructure 2. Lack of connectivity 3. Lack of availability of resources
Financial	1. Lack of budget/fund 2. Rewarding and incentives 3. Cost of OER applications
Pedagogical	1. Lack of pedagogical knowledge to apply OER in education 2. Lack of engagement 3. Lack of skills and training 4. Lack of intellectual property
Personal	1. Lack of time 2. Lack of interest and motivation 3. Lack of competence 4. Culture 5. Language

From the infrastructure dimension, [47] underlined that connectivity and access are challenges that limit OER adoption. In addition [70], conducted a study was conducted on sustaining OER, and as a result of this study, awareness and finding available sources are considered challenges that limit OER in adoption. [42] found that some of the OER challenges in Africa include internet infrastructure, equitable access, expanding resources, and building communities of education collaborators to develop and use OER.

From the financial dimension, it was found limited funding opportunities had been found to create and promote OER. [52] further stated that academicians who create their resources as OER do not receive any awards or citations for their work and also have difficulties in ensuring justice in the digitization, distribution, access, and sustainability of resources. In [37] it is stated that although the cost of OER applications has decreased, their applicability has not been realized.

From the pedagogical perspective, it is seen that several teachers and stakeholders lack the knowledge or skills to create OER. For instance [65], stated that several challenges limit OER adoption, including: difficulty finding the relevant OER; lack of computer skills to search for OER; lack of ability to adapt different OER; and lack of ability to interpret different OER licenses. [67] further pointed out that to facilitate OER adoption in Africa, more focus should be put on in-service OER training and teacher professional development. [63] underlined that lack of common understanding of OER as well as the needed competence and experience limit OER adoption. [51] emphasized that there are barriers to the operationalization of OER, and research participants have knowledge and understanding of OER but this has not been translated into active participation. The most prominent conclusion from these suggestions is that more OER training should be provided for the professional development of teachers in this regard.

From the personal perspective [43], pointed out that workload is a challenge for teachers in African universities; they are overloaded and do not have time to create OER. [48] also mentioned that lack of interest in creating and/or using OER is one of the challenges of OER adoption. [74] further pointed out that culture might also play a role in OER adoption in Africa, as most teachers believe that high-quality educational resources should not be free and created by anyone.

## 4. Conclusion

The aim of this systematic review was to summarize and provide insights into OER research in Africa. The results showed that varied research studies have been conducted and that they are on the increase. However, it is notable that most research is confined to certain countries and that there is not an even spread of research activities in all countries in the continent. From an overview of initiatives in Africa, the emphasis on OER creation and the importance of outside collaborators are clear. Thematically, most of the research focused on the role of OER in different aspects of education, followed by a focus on social justice. The systematic review concludes by identifying certain challenges that limit the adoption of OER and OEP. In this regard, issues around policies were highlighted in the corpus. The importance of infrastructure and financial aspects were also evident. Furthermore, challenges also related to pedagogical, technical, and personal dimensions in the corpus.

Several recommendations can be made based on the systematic review presented in this paper. In this regard, more research is necessary in countries where limited work has been done on this topic. Furthermore, a move away from the focus on OER creation to adoption and dissemination is advised. There is also a clear need for more initiatives driven and supported from within Africa. It is important that research is extended beyond general educational aspects and social justice. Thematically, aspects around challenges for OER, cultural and policy issues need further investigation. Finally, the identified challenges pose many opportunities for future research and interventions toward effective implementation of OER and OEP.

It should be noted that this study has several limitations that should be acknowledged and further researched. For instance, the findings might be limited to the search keywords, as well as the electronic databases used. However, despite these limitations, this study presents a solid ground related to OER in Africa, hence, contributing to achieving the UN Sustainable Development Goals (SDGs) connected to the use of OER, especially SDG #4 (Equity and high-quality education for all), which works as a backbone of some other SDGs, for example, Good Health (#3), Economic Growth (#8), and Reduced Inequality (#10).

## Supporting information

S1 Checklist Prisma 2009 checklist. (DOC)

## References

[1] Ayentimi D. T., & Burgess J. (2019). Is the fourth industrial revolution relevant to sub-Sahara Africa? *Technology analysis & strategic management*, 31(6), 641–652.

[2] Olivier J. (2020). Self-directed open educational practices for a decolonized South African curriculum: a process of localization for learning. *Journal of e-Learning and Knowledge Society*, 16(4), 20–28.

[3] UNESCO. (2019). Recommendation on Open Educational Resources (OER). Paris: UNESCO. Retrieved 13 May 2021, from http://portal.unesco.org/en/ev.php-URL_ID=49556&URL_DO=DO_TOPIC&URL_SECTION=201.html

[4] Ministry of Education (2014). Information Communications Technology (ICT) in Education and Training Policy—2014–2019. https://planipolis.iiep.unesco.org/sites/default/files/ressources/seychelles_ict_education_policy.pdf

[5] Arinto P.B., Hodgkinson-Williams C., King T., Cartmill T. & Willmers M. (2017). Research on Open Educational Resources for Development in the Global South: Project landscape. In Hodgkinson-Williams C. & Arinto P. B. (Eds.), Adoption and impact of OER in the Global South (pp. 3–26). Cape Town & Ottawa: African Minds, International Development Research Centre, Research on Open Educational Resources for Development. doi: 10.1371/journal.pone.0169883

[6] Commonwealth of Learning. (2017). REPORT MENA Regional Consultation on Open Educational Resources. Retrieved 05 January, 2020 from http://rcoer.col.org/uploads/2/2/8/4/22841180/mena_rcoer_report_18

[7] Huang R., Liu D., Tlili A., Gao Y., & Koper R. (2020). *Current state of open educational resources in the “belt and road” countries*. Springer.

[8] Yilmaz R. M., Topu F. B., &TakkaçTulgar A (2019). An examination of the studies on foreign language teaching in pre-school education: A bibliometric mapping analysis. *Computer Assisted Language Learning*, 1–24. 10.1080/09588221.2019.1681465

[9] Vogel R., & Masal D. (2015). Public leadership: A review of the literature and frame-work for future research. *Public Management Review*, 17(8), 1165–1189. 10.1080/14719037.2014.895031

[10] Dale B.G., Y.-Wu P., Zairi M., Williams A.R.T and Van der Wiele T. (2001). Total quality management and theory: An exploratory study of contribution, *Total quality management*, 12 (4),439–449.

[11] Balcı A., (2005). *Researches in Social Sciences*. Ankara: Pegem Press, pp.387.

[12] Harden A., & Thomas J. (2010). Mixed methods and systematic reviews: Examples and emerging issues. In Tashakkori A. & Teddlie C. (Eds.), *Sage handbook of mixed methods in social & behavioral research*, (pp. 749–774). SAGE.

[13] Marangunić N., & Granić A. (2015). Technology acceptance model: a literature review from 1986 to 2013. *Universal Access in the Information Society*, 14(1), 81–95.

[14] Webster J., & Watson R.T. (2002). Analyzing the past to prepare for the future: writing a literature review. MIS Q. 26(2), xiii–xxiii.

[15] He Q., Wang G., Luo L., Shi Q., Xie J., & Meng X. (2017). Mapping the managerial areas of Building Information Modeling (BIM) using scientometric analysis. *International Journal of Project Management*, 35(4), 670–685.

[16] Oraee M., Hosseini M. R., Papadonikolaki E., Palliyaguru R., & Arashpour M. (2017). Collaboration in BIM-based construction networks: A bibliometric-qualitative literature review. *International Journal of Project Management*, 35(7), 1288–1301.

[17] Tlili, A., Burgos, D., Altınay, F., Altınay, Z., Huang, R., & Jemni, M. (2021a). Remote Special Education during COVID-19: A Combined Bibliometric, Content and Thematic Analysis. In 2021 International Conference on Advanced Learning Technologies (ICALT) (pp. 325–329). IEEE.

[18] Moher D., Liberati A., Tetzlaff J., & Altman D. G. (2010). Preferred reporting items for systematic reviews and meta-analyses: the PRISMA statement. *Int J Surg*, 8(5), 336–341. doi: 10.1016/j.ijsu.2010.02.007 20171303

[19] Given L. M. (2008). *The Sage encyclopedia of qualitative research methods*. Sage.

[20] Foster R. L. (1997). Addressing epistemologic and practical issues in multimethod research: A procedure for conceptual triangulation. Advances in Nursing Science, 20(2), 1–12. Retrieved from https://journals.lww.com/advancesinnursingscience/pages/default.aspx doi: 10.1097/00012272-199712000-00002 9398934

[21] Van Eck N. J., & Waltman L. (2010). Software survey: VOSviewer, a computer program for bibliometric mapping. *Scientometrics*, 84(2), 523–538. doi: 10.1007/s11192-009-0146-3 20585380 PMC2883932

[22] Harzing A. W., & Alakangas S. (2016). Google Scholar, Scopus and the Web of Science: a longitudinal and cross-disciplinary comparison. *Scientometrics*, 106(2), 787–804.

[23] Tlili A., Burgos D., Huang R., Mishra S., Sharma R. C., & Bozkurt A. (2021b). An Analysis of Peer-Reviewed Publications on Open Educational Practices (OEP) from 2007 to 2020: A Bibliometric Mapping Analysis. Sustainability, 13(19), 10798.

[24] Cohen J. (1960). A coefficient of agreement for nominal scales. *Educational and Psychological Measurement*, 20(1), 37–46. 10.1177/001316446002000104

[25] Miller A. (2015). Games centered approaches in teaching children & adolescents: Systematic review of associated student outcomes. *Journal of teaching in physical education*, 34(1), 36–58.

[26] Spolaôr N., &Benitti F. B. V. (2017). Robotics applications grounded in learning theories on tertiary education: A systematic review. *Computers & Education*, 112, 97–107.

[27] Tlili A., Nascimbeni F., Burgos D., Zhang X., Huang R., & Chang T. W. (2020). The evolution of sustainability models for Open Educational Resources: insights from the literature and experts. *Interactive Learning Environments*, 1–16.

[28] Britten N., Campbell R., Pope C., Donovan J., Morgan M., & Pill R. (2002). Using meta ethnography to synthesize qualitative research: A worked example. *Journal of Health Services Research and Policy*, 7(4), 209–215. doi: 10.1258/135581902320432732 12425780

[29] Zawacki-Richter O., Bäcker E. M., & Vogt S. (2009). Review of distance education research (2000 to 2008): Analysis of research areas, methods, and authorship patterns. *International Review of Research in Open and Distributed Learning*, 10(6), 21–50.

[30] Bateman P., Lane A., & Moon R. (2012). Out of Africa: a typology for analysing open educational resources initiatives. *Journal of Interactive Media in Education*, 2, 1–14.

[31] Brereton P., Kitchenham B. A., Budgen D., Turner M., & Khalil M. (2007). Lessons from applying the systematic literature review process within the Software Engineering domain. *Journal of Systems and Software*, 80, 571–583.

[32] Wolfenden F. (2008). The TESSA OER Experience: Building Sustainable Models of Production and User Implementation. *Journal of Interactive Media in Education*. 10.5334/2008-3

[33] Thakrar J., Wolfenden F., & Zinn D. (2009). Harnessing open educational resources to the challenges of teacher education in Sub-Saharan Africa. *International Review of Research in Open and Distributed Learning*, 10(4).

[34] Adanu R. M. K., Adu-Sarkodie Y., Opare-Sem O., Nkyekyer K., Donkor P., Lawson A., et al. (2010). Electronic learning and open educational resources in the health sciences in Ghana. *Ghana Medical Journal*, 44(4). doi: 10.4314/gmj.v44i4.68910 21416051 PMC3052833

[35] Gakindi Jr, M. (2010). Information access needs of satellite campuses in Kenya-Can OER close the gap? The Case of Moi University Nairobi Campus (Doctoral dissertation, Tallinn University (Estonia).

[36] Tagoe, N., Donkor, P., Adanu, R., Opere-Sem, O., Engleberg, C., & Lawson, A. (2010). Beyond the first steps: Sustaining Health OER initiatives in Ghana. Paper presented at the 7th Annual Open Education Conference, Barcelona. Retrieved 2 June 2021, from http://openaccess.uoc.edu/webapps/o2/handle/10609/4849

[37] Hodgkinson-Wiliams, C., & Donnelly, S. (2010). Sustaining OER at the University of Cape Town: Free, but not cheap. In Open Ed 2010 Proceedings. Barcelona: UOC, OU, BYU.

[38] Sapire I., & Reed Y. (2011). Collaborative design and use of open educational resources: A case study of a mathematics teacher education project in South Africa. *Distance Education*, 32(2), 195–211.

[39] Harley K. (2011). Insights from the Health OER inter-institutional project. *Distance Education*, 32(2), 213–227.

[40] Essuman, S. O., & Asante, K. (2012). The student-teacher in the 21st century classroom and the use of Open Educational Resources: The case of early childhood case of early interns of the university of education, Winneba. In EDULEARN12 Proceedings (pp. 839–852). IATED.

[41] Wolfenden F., Buckler A., &Keraro F. (2012). OER adaptation and reuse across cultural contexts in Sub Saharan Africa: Lessons from TESSA (Teacher Education in Sub Saharan Africa). *Journal of Interactive Media in Education*, 10.5334/2012-03

[42] Wright C. R., &Reju S. A. (2012). Developing and deploying OERs in sub-Saharan Africa: Building on the present. *International Review of Research in Open and Distributed Learning*, 13(2), 181–220.

[43] Mays, T. J., & Krull, G. (2012) Introducing OER for teacher development in African universities. *In Open Distance Learning (ODL) conference*.

[44] Mibey R. K. (2012). Strengthening science and technological capacity in Africa: Engaging women and youth. Accessed from: https://repository.ruforum.org/system/tdf/Mibey.pdf?file=1&type=node&id=32397&force=

[45] Percy, T., & Van Belle, J. P. (2012). Exploring the barriers and enablers to the use of open educational resources by university academics in Africa. In IFIP International Conference on Open Source Systems (pp. 112–128). Springer, Berlin, Heidelberg.

[46] Murphy P., & Wolfenden F. (2013). Developing a pedagogy of mutuality in a capability approach: Teachers’ experiences of using the Open Educational Resources (OER) of the teacher education in sub-Saharan Africa (TESSA) programme. *International Journal of Educational Development*, 33(3), 263–271.

[47] Lesko I. (2013). The use and production of OER & OCW in teaching in South African higher education institutions (Case study). *Open Praxis*, 5(2), 103–121. doi: 10.3390/nano6110203 28335331 PMC5245737

[48] Mtebe J. S., &Raisamo R. (2014a). Investigating perceived barriers to the use of open educational resources in higher education in Tanzania. *International Review of Research in Open and Distributed Learning*, 15(2), 43–66.

[49] Mtebe J. S., &Raisamo R. (2014b). Challenges and instructors’ intention to adopt and use open educational resources in higher education in Tanzania. *International review of research in open and distributed learning*, 15(1), 249–271.

[50] Kandiero, A. (2015). Educators’ challenges and behavioural intention to adopt open educational resources: the case of Africa University, Zimbabwe (Master’s thesis, University of Cape Town).

[51] De Hart K. L., Chetty Y. B., & Archer E. (2015). Uptake of OER by staff in distance education in South Africa. *International Review of Research in Open and Distributed Learning*, 16(2), 18–45.

[52] Raju R., Adam A., & Powell C. (2015). Promoting open scholarship in Africa: Benefits and best library practices. *Library Trends*, 64(1), 136–160.

[53] Mukhtar, D.M. I. (2016). Professionalism and open educational resources (OER) in Africa: Teacher professional and professionalism Development via OER. Accessed from: https://globalacademicgroup.com/journals/the%20intuition/MOHAMMED%201.pdf

[54] Welch T., & Glennie J. (2016). Open Educational Resources for Early Literacy in Africa: The role of the African storybook initiative. In Miao F., Mishra S., & McGreal R. (Eds.), *Open educational resources*: *Policy*, *costs*, *transformation* (pp.195–210). UNESCO and Commonwealth of Learning.

[55] Cox G., & Trotter H. (2016). Institutional culture and OER policy: How structure, culture, and agency mediate OER policy potential in South African universities. *International Review of Research in Open and Distributed Learning*: *IRRODL*, 17(5), 147–164.

[56] Jung I., & Hong S. (2016). Faculty members’ instructional priorities for adopting OER. *The International Review of Research in Open and Distributed Learning*, 17(6).

[57] Muganda C. K., Samzugi A. S., & Mallinson B. J. (2016). Analytical insights on the position, challenges, and potential for promoting OER in ODeL institutions in Africa. *International review of research in open and distributed learning*, 17(4), 36–49.

[58] Mays T. J. (2017). Mainstreaming Use of Open Educational Resources (OER) in an African Context. *Open Praxis*, 9(4), 387–401.

[59] Cox G., & Trotter H. (2017). An OER framework, heuristic and lens: Tools for understanding lecturers’ adoption of OER. *Open Praxis*, 9(2), 151–171.

[60] Hodgkinson-Williams C. & Arinto P.B. (2017). *Adoption and impact of OER in the Global South*. Cape Town: African Minds, International Development Research Centre & Research on Open Educational Resources.

[61] Hodgkinson-Williams C., Arinto P. B., Cartmill T., & King T. (2017). Factors influencing open educational practices and OER in the Global South: Meta-synthesis of the ROER4D project. In Hodgkinson-Williams C. & Arinto P. B. (Eds.), *Adoption and impact of OER in the Global South* (pp. 27–68). Cape Town & Ottawa: African Minds, International Development Research Centre, Research on Open Educational Resources for Development.

[62] Hodgkinson-Williams C. A., & Trotter H. (2018). A Social Justice Framework for Understanding Open Educational Resources and Practices in the Global South. *Journal of Learning for Development*, 5(3), 204–224.

[63] Pete J., Mulder F., Neto J. O., & Omollo K. (2018). Differentiation in Access to, and the Use and Sharing of (Open) educational resources among students and lecturers at technical and comprehensive Ghanaian Universities. *Open Praxis*, 10(4), 405–421.

[64] King M., Pegrum M., & Forsey M. (2018). MOOCs and OER in the Global South: Problems and potential. *The International Review of Research in Open and Distributed Learning*, 19(5).

[65] Nkwenti M. N., & Abeywardena I. S. (2019). OER Mainstreaming in Cameroon: Perceptions and Barriers. *Open Praxis*, 11(3), 289–302.

[66] Chikuni P. R., Cox G., & Czerniewicz L. (2019). Exploring the Institutional OER Policy Landscape in South Africa: Dominant Discourses and Assumptions. *International Journal of Education and Development using Information and Communication Technology*, 15(4), 165–179.

[67] Otto D. (2019). Adoption and Diffusion of Open Educational Resources (OER) in Education: A Meta-Analysis of 25 OER-Projects. *International Review of Research in Open and Distributed Learning*, 20(5), 122–140.

[68] Loglo F. S., & Zawacki-Richter O. (2019). Exploring OER Awareness and Engagement of Academics from a Global South Perspective–a Case Study from Ghana. *ZeitschriftfürHochschulentwicklung*, 14(2), 15–42.

[69] Mays T. (2020). Open Educational Resources in South Africa. In Huang R., Liu D., Tlili A., Gao Y., & Koper R. (Eds.), Current State of Open Educational Resources in the “Belt and Road” Countries (pp. 195–212). Springer Singapore. 10.1007/978-981-15-3040-1_11

[70] Zaid Y. A., & Alabi A. O. (2020). Sustaining Open Educational Resources (OER) initiatives in Nigerian Universities. Open Learning: *The Journal of Open, Distance and e-Learning*, 1–18.

[71] Tlili A., Jemni M., Khribi M. K., Huang R., Chang T. W., & Liu D. (2020). Current state of open educational resources in the Arab region: an investigation in 22 countries. *Smart Learning Environments*, 7, 1–15.

[72] Buckler A., Stutchbury K., Kasule G., Cullen J., & Kaije D. (2021). What Prevents Teacher Educators from Accessing Professional Development OER and MOOC? Storytelling and Professional Identity in Ugandan Teacher Colleges.

[73] Wiche H. I., & Ogunbodede K. F. (2021). Awareness and Use of Open Educational Resources by Library and Information Science Students of Ignatius Ajuru University of Education, Rivers State, Nigeria.

[74] Tlili A., Ofosu S., & Zhang J. (2021c). Pedagogical beliefs, teaching practices and use of open educational resources in the Republic of Ghana. *Interactive Learning Environments*, 1–13.

[75] Bliss T. J., & Smith M. (2017). A Brief History of Open Educational Resources. In Jhangiani R. S. & Biswas-Diener R. (eds.), *Open*: *The philosophy and practices that are revolutionizing education and science* (pp. 9–27), Ubiquity Press.

[76] Ngimwa P., & Wilson T. (2012). An empirical investigation of the emergent issues around OER adoption in Sub-Saharan Africa. Learning, *Media and Technology*, 37(4), 398–413.

[77] William and Flora Hewlett Foundation. (2021). *Open Education*. Retrieved 2 June 2021, from https://hewlett.org/strategy/open-education/.

[78] UNESCO. (2012). The Paris OER Declaration 2012. https://en.unesco.org/oer/paris declaration

[79] Tlili A., Essalmi F., Jemni M., Kinshuk., & Chen N. S. (2016). Role of personality in computer based learning. *Computers in Human Behavior*, 64, 805–813.

[80] Atenas J., Havemann L., & Priego E. (2015). Open data as open educational resources: Towards transversal skills and global citizenship. *Open praxis*, 7(4), 377–389.

[81] Cronin C. (2017). Openness and praxis: Exploring the use of open educational practices in higher education. *International Review of Research in Open and Distributed Learning* 18(5), 15–34.

[82] Van Eck N.J., & Waltman L. (2018). VOSviewer Manual. Univeristeit Leiden, Leiden. Available from: http://www.vosviewer.com/documentation/Manual_VOSviewer_1.6.8.pdf

[83] UNESCO (2016). Every Child Should Have a Textbook (GEMR Policy Paper 23). Retrieved 2 June 2021, from https://en.unesco.org/gem-report/every-child-should-have-textbook.

